# Spatial Distribution and Enrichment Dynamics of Foodborne Norovirus in Oyster Tissues

**DOI:** 10.3390/foods13010128

**Published:** 2023-12-29

**Authors:** Mao Mao, Zilei Zhang, Xuchong Zhao, Haoran Geng, Liang Xue, Danlei Liu

**Affiliations:** 1 State Key Laboratory of Applied Microbiology Southern China, Institute of Microbiology, Guangdong Academy of Sciences, Guangzhou 510070, China; mimi12770415@163.com (M.M.); zhangzilei@shcc.edu.cn (Z.Z.); 2 Shanghai International Travel Healthcare Center, Shanghai Customs District P. R. China, Shanghai 200335, China; 3 Inspection and Quarantine Technology Communication Department, Shanghai Customs College, Shanghai 201204, China; 4 Jinan Center for Disease Control and Prevention, Jinan 250021, China; zhaoxuchong@163.com; 5 Shanghai-MOST Key Laboratory of Health and Disease Genomics, NHC Key Lab of Reproduction Regulation, Shanghai Institute for Biomedical and Pharmaceutical Technologies, Fudan University, Shanghai 200237, China; hrgeng20@fudan.edu.cn

**Keywords:** norovirus contamination, oyster tissues, temporal dynamics, detection strategies, foodborne infections

## Abstract

The prevalence of norovirus in oysters poses a significant threat to food safety, necessitating a comprehensive understanding of contamination patterns. This study explores the temporal dynamics of norovirus distribution in various oyster tissues over a contamination period ranging from 6 to 96 h. Four tissues—the gill, palp, digestive gland, and stomach—were subjected to systematic monitoring using RT-qPCR for absolute quantification. Results revealed rapid norovirus detection in all tissues six hours post-contamination, with subsequent variations in detection rates. Gill and digestive gland tissues exhibited a peak in detection at 12–24 h, aligning with the oyster’s gastrointestinal circulatory system. The digestive gland, distinguished by specific enrichment and adsorption capabilities, demonstrated the highest virus concentration at 48 h. In contrast, the stomach displayed a reemergence of norovirus. Beyond 72 h, detection remained exclusive to the digestive gland, with Ct values comparable to earlier time points. At 96 h, a limited amount of norovirus was detected in the digestive gland, emphasizing the importance for timely monitoring. In addition to providing critical insights into optimal detection strategies, these findings highlight the time-related characteristics of norovirus contamination in oysters. The study identifies the digestive gland as a key target for reliable monitoring, providing valuable data to improve protocols for reducing hazards associated with oyster consumption and foodborne norovirus infections. This research contributes to the understanding of norovirus dynamics in oyster tissues and reinforces current efforts aimed at ensuring food safety and public health.

## 1. Introduction

Foodborne noroviruses, commonly referred to as noroviruses (NoV), are a significant global public health concern due to their association with foodborne illnesses and outbreaks. These highly contagious viruses are a leading cause of acute gastroenteritis, affecting individuals of all ages. Large-scale outbreaks in schools and communities can lead to the closure of growing areas and the recall of shellfish products, resulting in serious life threats and huge economic losses [1]. The global impact of NoV is growing, with an estimated 684 million cases and 212,000 deaths occurring each year [2]. NoVs cause acute gastroenteritis in humans for the following reasons: NoVs are usually associated with foodborne and waterborne transmission; NoVs are highly stable in the environment, and only 18 viral particles could cause disease; there are currently no antiviral drugs specific for NoVs [3]. The primary mode of transmission is the fecal–oral route, which can occur through contaminated water, person-to-person contact, and, importantly for this study, consumption of contaminated food, particularly seafood. Based on genetic differences found in the capsid protein, the NoV is currently classified into at least 49 genotypes within 10 genogroups (GI to GX) [4]. Acute gastroenteritis cases in humans are associated with two epidemiologically dominant genogroups, GI and GII, that are further subdivided into nine genotypes and 27 genotypes, respectively. According to global surveillance data, several NoV genotypes are responsible for outbreaks, with GII.4 constituting the majority [5,6].

In recent years, significant attention has been given to NoV outbreaks that are linked to the consumption of raw or undercooked oysters. Oysters, filter-feeding bivalve mollusks, can bioaccumulate NoV from contaminated water sources [7]. These sources include effluent from wastewater treatment facilities, combined sewer/stormwater overflows, malfunctioning septic tanks, and recreational and commercial fishing vessels [8]. There are varieties of oysters all over the world, such as *Crossostrea gigas*, *Crassostrea gigas angulate*, *Crassostrea hongkongensis*, *Ostrea rivularis Gould*, and *Crassostrea sikamea*. The Pacific oyster (*Crassotrea gigas*), naturally distributed around Japan, China, and Korea, is widely now an important cultivated oyster species worldwide [7]. It is becoming increasingly evident that viruses interact with oysters, and the dynamics in oysters after infection have been strongly linked to massive oyster mortality and human illnesses, such as gastroenteritis and NoV outbreaks. In outbreaks caused by shellfish, genotyping of norovirus in stool and oyster samples may reveal discrepancies. This raises the question of what role the oyster played and could be overcome by analyzing viral diversity in greater detail [9].

Currently, there are numerous issues linked to viral contamination. Over the past 10 years, more than 40% of RASFF (Rapid Alert System for Food and Feed) notifications were related to NoV detection in oysters [10]. Studies from various countries have consistently shown a high prevalence of NoV in commercially harvested oyster samples, raising significant concerns regarding the contamination of oysters with NoV [11]. Chassaing et al. conducted a meta-analysis of publications on NoV-related foodborne outbreaks from 1993 to 2019 and found that bivalve molluscan shellfish accounted for 53% of the implicated food sources [12]. For perspective, the prevalence of NoV in oysters from other regions is reported in the range of <2% in Australia, 9% in France, 3.9 to 20% in the USA, 16.9% in China, 32.1% in Spain, and up to 71.6% in the United Kingdom [13]. Our previous research entailed a year-long investigation into oyster contamination in aquaculture farms and revealed that 16.9% (60/356) of the collected oyster samples tested positive for NoV [14]. A systematic review and meta-analysis indicated a substantial burden of NoV in shellfish worldwide, with GII.4 being the predominant genotype [15]. These findings emphasize the widespread prevalence of NoV contamination in oyster samples and highlight the need for further research to elucidate the dynamics of norovirus distribution in oyster populations.

The detection of NoV in both food and environmental samples remains a significant challenge, primarily due to the complex composition of sample tissues and the low concentration of the virus present. Current human NoV cell culture systems are complicated and do not allow infectious NoV detection at levels found in contaminated foods [16]. Similarly to other viruses, detecting and monitoring NoV RNA encompasses several steps, including sample collection, concentration and enrichment, laboratory assay, and data normalization and interpretation. In the absence of methods to determine the viral infectivity of NoVs in food samples, foodborne viruses are detected mostly by molecular methods. Molecular techniques are currently the gold standard for discovering NoVs, including reverse transcription-polymerase chain reaction (RT-PCR), droplet digital RT-PCR (RT-ddPCR), and quantitative real-time RT-PCR. Among those methods, RT-qPCR has emerged as a valuable tool in the detection and quantification of NoVs due to its high sensitivity and specificity, as well as its low risk of carry-over contamination [17]. As of now, various commercially available RT-qPCR assays demonstrate the sensitivity of real-time RT-PCR at approximately 10–50 genome copies per reaction for NoV GI and 1–300 genome copies per reaction for NoV GII [18]. For accurate genotype identification of positive samples, RT-PCR and nested RT-PCR were used, followed by amplicon sequencing.

The adoption of RT-qPCR for NoV detection aligns with the international standard ISO 15216 [19], therefore enhancing the credibility and applicability of the detection method. Moreover, while the absence of genomes is necessarily a sign of that the corresponding infectious viruses are absent, ISO 15216, when strictly applied, imposes a theoretical limit of detection (LOD) of greater than 18 particles, usually defined as the minimal NoV infective dose [20]. In addition to using of RT-qPCR, several studies have focused on optimizing and comparing the enrichment processes used in the preprocessing of oyster tissues, aimed at enhancing the efficiency and accuracy of detecting NoV in oyster samples [19,21,22,23]. Meanwhile, differential detection rates of NoV have been shown within distinct oyster tissues, shedding light on the complex dynamics of NoV distribution and persistence within oyster organisms [24,25]. Therefore, understanding the distribution and prevalence of NoV in oysters is crucial for improving detection rate and reducing the false-positive rate.

There have been numerous studies investigating shellfish virus contamination. However, most of the above studies focused on a specific country, a certain period, and limited species types. This is not conducive to a comprehensive understanding of the overall contamination of NoV in shellfish worldwide. In this study, we explored the spatial dynamic distribution of NoV in different oyster tissues by constructing an artificially contaminated oyster model to clarify the in vivo circulation path and excretion time of NoV. Simultaneously, the generated data can support the development of virus detection preprocessing methods for oysters and contribute to the design of depuration strategies.

## 2. Materials and Methods

### 2.1. Artificial Contamination Oysters with NoV

All experiments were performed in a biosafety level 2 (BSL-2) containment laboratory using standard BSL-2 work practices. In this study, viral titers were measured using the RT-qPCR method. The stool sample with the highest copy number for NoV was about 1.42 × 10^7^ genome copies per mL.

The oysters (*Crassotrea gigas*) were obtained from Zhejiang Province, China. After surface cleaning, oysters were transferred into 20 L of clean water with 1.8% sea salt (Yier Biological Engineering Co., Ltd., Guangzhou, China), dissolved to simulate a marine environment. An air pump (Sunsun group co., Ltd., Zhoushan, China) supplied oxygen continuously. Before the experiment, the oysters were immersed in salt water for 24 h. To ensure the cleanliness of the oysters used in the experiments, three oysters from each group were shucked, homogenized, and tested for NoV before being artificially contaminated. After 24 h of acclimation, all fresh oysters were randomly divided into two groups (16–18 oysters per group). Each group was exposed to 4 L of water with 1.8% sea salt under oxygenation with 1.0 mL NoV-positive GII.4 stool samples (final titer around 10^6^ genome copies/liter) at room temperature to simulate a NoV-polluted environment. The water was circulated for 96 h continuously, and the experiment lasted for 5 days. During the whole experiment, oyster mortality was checked daily, and dead oysters were promptly removed. Two biological replicates were taken randomly at each time point from each group to ensure four parallel oyster samples for each time point.

### 2.2. Sample Collection and Processing Procedures

Oyster samples were collected at specific time points (6 h, 12 h, 24 h, 48 h, 72 h, 96 h) for further analysis. Samples were tested for NoV under ISO 15216 [19]. Briefly, the oysters’ external surfaces were thoroughly washed and sterilized with alcohol before being opening. And then the oysters were dissected, and the digestive glands, gills, stomach, and labial palps were isolated. The latest sterile disposable plasticware and razor blades were used to process each sample. A tissue weighing 1.0 ± 0.1 g was measured and transferred to a 1.5 mL centrifuge tube. Proteinase K (1.0 mL, 3000 U/L) was added, and the samples were incubated for 1 h at 37 °C with stirring at 200 rpm. As a follow-up step, a 15 min incubation was conducted in a water bath at 60 °C. After centrifugation at 13,000× *g* for 5 min at 4 °C, the supernatant was carefully transferred to a clean tube. To extract viral RNA, the HiPure Viral RNA Kit (Magen, Guangzhou, China) was used following the manufacturer’s guidelines. Viral RNA extraction was performed with 140.0 μL of supernatant, and elution was collected in 20.0 μL of DNase/RNase-free water. The extracted RNA was tested immediately, and the remaining RNA was stored at −80 °C for further molecular analysis.

### 2.3. Quantitative Real-Time PCR (RT-qPCR) for NoV

According to our previous report, duplicate one-step RT-qPCR assays for GI and GII NoV were conducted in a single tube using the Bio-Rad CFX96 qPCR machine [26]. Each 20.0 μL reaction mixture consisted of 6.0 μL of viral RNA samples. The cycling conditions were as follows: 5 min at 42 °C, and then 15 s at 95 °C, followed by 40 cycles consisting of 5 s at 95 °C and 10 s at 60 °C. Fluorescence was read at the end of each 60 °C extension step. The PCR cycle threshold (Ct) values were determined with Biorad CFX96 Manager Software version 3.1, which utilizes amplification-based threshold determination with default settings. A cycle threshold Ct value of ≤40 and a signal demonstrating a substantial exponential increase were considered positive samples. NoV GII genome copy numbers per reaction were calculated using an RT-qPCR standard curve, as previously described, and then converted to genome copies per gram of the tissues. Statistical evaluation was performed using Microsoft Excel and GraphPad Prism 7 (GraphPad Software, USA, www.graphpad.com).

## 3. Results

### 3.1. Dynamics of NoV Contamination in Oyster Tissues

**Figure 1:**
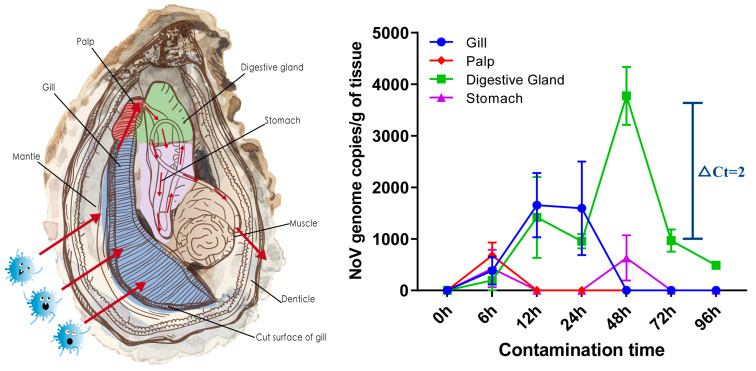
Schematic illustration of oyster tissue structure (**left**) and dynamics in the line chart of norovirus contamination in oyster tissues (**right**). Each tissue is colored from left to right in the same order. The bar graph presents the mean and STDEV from four parallel samples, with superimposed symbols at the mean and a connecting line. Oyster’s anatomical structure and its gastrointestinal circulatory system were adapted with permission from Ref. [27]. 2021, Elsevier. The arrow indicates the direction of the virus’s movement.

In this comprehensive exploration of NoV contamination dynamics within oyster tissues, our focus was on the intricate interplay between the oyster’s anatomical structure and its gastrointestinal circulatory system. A strategic selection of four tissues—the gill, palp, digestive gland, and stomach—was chosen to elucidate the internal circulation pathway and timeframe of NoV excretion within contaminated oysters (Figure 1) [27]. To achieve this, we employed RT-qPCR technology for precise quantitative analysis across tissues, with the original data available in Supporting Information Table S1. Results revealed a notable progression in NoV detection, with positive signals identified in all four tissues after the initial contamination stage at 6 h. After 12 to 24 h, only the gill and digestive gland tissues showed evidence of infection, with the gill tissue exhibiting a greater viral quantity than the digestive gland. The 48 h mark witnessed the virus reaching its zenith concentration in the digestive gland, coinciding with the absence of detectable virus in the gill tissue but reappearing in the stomach. A fascinating feature of this pattern is that it aligns with that of oyster tissues’ digestive circulation, suggesting a specific enrichment and adsorption role for NoV in the gastrointestinal system. Beyond 72 h, only the digestive gland tissue displayed detectable NoV levels, with Ct values comparable to those at 12 and 24 h post-contamination, differing by a modest 2.3 Ct value from the peak at 48 h. At 96 h, only a small amount of NoV was detected in the digestive gland tissue. It is, however, insufficient to ascertain the virus’s infectivity at this time due to the limited cultivation system for NoV.

### 3.2. Relationship between Sampling Sites and the Detection Rate

**Figure 2:**
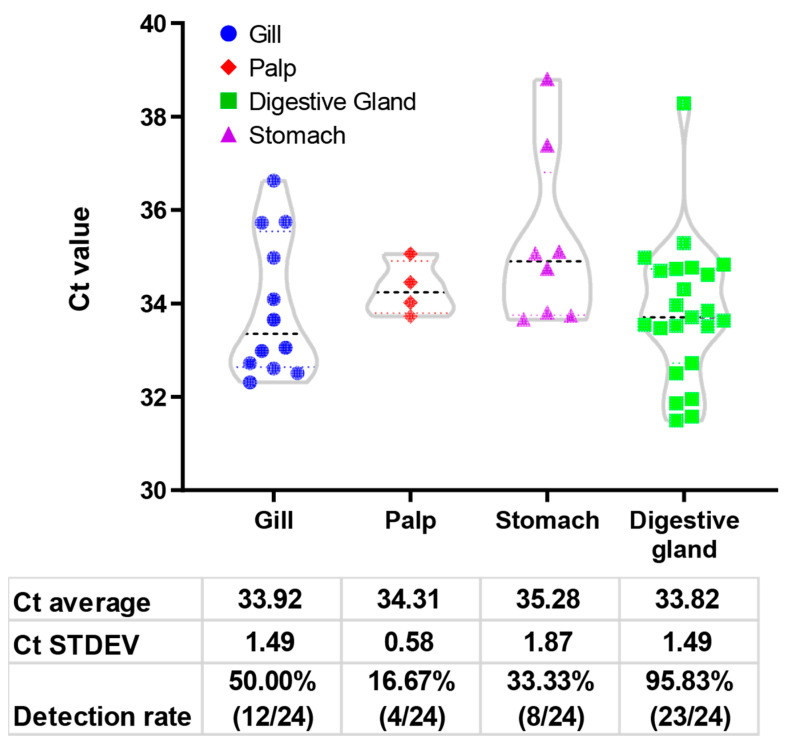
Violin plot of the relationship between sampling sites and Ct value. Each dot represents an organ from an oyster. Each compartment of the tissue is represented by a distinct color. Below are the density plots, and the corresponding tables highlight the calculated mean and standard deviation (STDEV) for each distribution. The mean and STDEV were calculated for positive samples from each issue group.

The study delved into the relationship between various sampling sites and the detection efficiency of NoV, revealing noteworthy variations in detection rates across different tissues (Figure 2). The average Ct values demonstrated distinct trends, registering 33.92 for the gill, 34.31 for the palp, 35.28 for the stomach, and 33.82 for the digestive gland. Standard deviations (Ct STDEV) indicated larger levels of variability in the gill and digestive gland tissues, with values of 1.49 compared to the palp (0.58) and stomach (1.87). However, the digestive gland exhibited outstanding performance with a detection rate of 95.83% (23/24). In contrast, the gill, palp, and stomach showed lower detection rates of 50.00% (12/24), 16.67% (4/24), and 33.33% (8/24), respectively. This underscores the digestive gland as the most reliable site for NoV detection, as detection rates in all tissues, excluding the digestive gland, were below the 50% threshold. The observed variability in detection rates underscores the importance of strategic tissue selection for NoV detection in oysters.

### 3.3. Relationship between Contamination Time and Detection Rate

**Figure 3:**
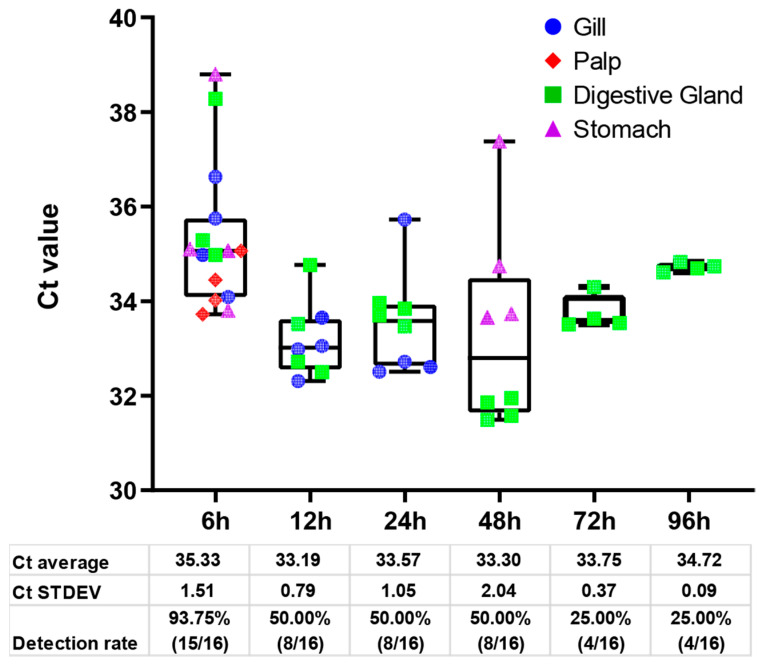
Boxplot diagram of the relationship between contamination time and detection rate. Each of the dots represents an organ from an oyster. Each tissue compartment is represented by a distinct color. Below are the density plots, and the corresponding tables highlight the calculated mean and standard deviation (STDEV) for each distribution. The mean and STDEV were calculated for positive samples of each time point group.

The investigation explored the nuanced interplay between contamination time and NoV detection rate in oysters, revealing distinctive patterns through statistical analysis (Figure 3). At an early contamination time of 6 h, a robust detection rate of 93.75% (15/16) was observed, with an average Ct value of 35.33 and a standard deviation (Ct STDEV) of 1.51. As contamination progressed for 12 and 24 h, the detection rate remained stable at 50.00% (8/16), accompanied by average Ct values of 33.19 and 33.57, respectively, indicating a sustained level of contamination. However, at 48 h, a slight decrease in detection rate to 50.00% (8/16) was coupled with an increased Ct STDEV of 2.04, indicating increased variability in detection levels. Subsequently, at 72 and 96 h, detection rates declined to 25.00% (4/16), with corresponding average Ct values of 33.75 and 34.72 and notably lower Ct STDEV values of 0.37 and 0.09, respectively. Based on these findings, NoV detection in oysters is highly dependent on the duration of contamination, with a high detection rate initially descending over time. Considering the variability in Ct values and detection rates, timely monitoring is essential to accurately assess the level of NoV contamination in oysters.

## 4. Discussion

The periodic monitoring of viral pathogens, such as NoV, is essential for determining infection status in the general population [15]. A gold standard for NoV detection has thus emerged in the form of RT-qPCR assays, recognized for their unparalleled sensitivity and ability to identify genetically diverse NoV strains [4]. In view of the fact that culture methods for NoV are not applicable to food matrices, the ISO 15216 approach relies instead on the method of RT-qPCR for detecting viral genomes, rather than the quantification of infectious viruses [19]. It should be noted that although the methods outlined in ISO 15216 are widely accepted for virus recovery and quantification from food surfaces, their applicability to the complex pretreatment matrices of food requires further assessment.

In order to mitigate the impact of food matrices on NoV detection, we investigated the spatial dynamic distribution of NoV in different oyster tissues. NoV contamination in oyster tissues was discussed in a nuanced manner based on our findings, including the impact of tissue-specific enrichment and the possibility of long-term persistence of NoV in digestive gland tissues. It can be concluded from our results that the digestive gland is the optimal target for NoV detection. Due to its consistency and high detection rate, the digestive gland makes an ideal target for NoV surveillance; it provides valuable insights for fine-tuning sampling strategies and improving surveillance progress accuracy. Timely and consistent monitoring plays a crucial role in accurately measuring the extent of NoV contamination in oysters, given the observed variability in Ct values and detection rates. Additionally, the preprocessing steps outlined in the existing ISO methods demonstrate stability, and prompt testing upon sample collection holds the potential to enhance overall detection efficiency. This temporal perspective enhances the understanding of NoV dynamics in oyster populations and aids in the development of effective monitoring and mitigation strategies.

Detection methods for NoV in food samples continue to face challenges in effectively separating the virus from complex food matrices. A critical step to resolving the bottleneck previously mentioned is to identify where NoV is distributed in oyster tissues, what binding sites it uses, and what mechanisms it uses for adsorption. NoVs persist in oyster tissues for extended periods through binding to specific antigens in the gill and digestive gland tissue. Immunohistochemical experiments have further illustrated the enrichment of NoV viral particles and constructed virus-like particles (VLPs) in various oyster tissues [28]. Research has also identified substances in oyster tissues that can be recognized by monoclonal antibodies targeting histo-blood group antigens (HBGAs) [29,30], with A-type HBGAs particularly implicated in the adsorption of NoV [31,32]. In prior investigations, our team employed a GII.4-type NoV bacterial cell surface display system to capture, identify, and validate protein ligands specific to NoV binding in oyster tissues. We observed that oyster heat shock protein 70 (HSP70) is a candidate vital ligand for the specific binding of NoV in oyster tissues, providing a better understanding of NoV attachment and transmission in oysters [33]. Through the comprehensive investigation of the spatial distribution and enrichment dynamics of NoV within various oyster tissues in this project, we provide valuable support and direction for subsequent studies focused on specific adsorption sites and target selection. This research contributes essential insights to advance the understanding of NoV interactions within oyster tissues, addressing a pivotal aspect in the refinement of NoV detection strategies.

The enrichment of viruses in oyster tissues is primarily attributed to physical filtration during bivalve feeding. However, the accumulation of NoV in oyster tissues may involve more specific interactions, significantly complicating the purification process [34]. Heat treatment, while effective in inactivating the virus, compromises shellfish texture and flavor, thereby diminishing their sensory qualities and consumer value [35]. Purification procedures entail placing harvested shellfish in clean water to naturally reduce bacterial and viral content through the digestion and excretion processes. This method is widely employed in the global shellfish industry to eliminate or diminish the contamination of pathogens. Nevertheless, these purification methods exhibit limited efficacy in addressing contamination concerns. The depuration process can eliminate certain viruses such as feline calicivirus, but others, such as NoV, can remain persistent in oyster tissues for long periods [36].

Consequently, understanding the dynamics of NoV enrichment in certain tissues, including the digestive gland, will help target purification efforts and contribute to the development of more efficient decontamination processes for oysters. There may also be persistent latent infections that cannot be detected using PCR methods due to low viral loads. A 10- to 100-fold increase may occur due to the small volume analyzed by RT-qPCR, the low rate of recovery during genome extraction, and the presence of RT-qPCR inhibitors. Thus, except in some specific cases (i.e., in cases with very high levels of pollution or artificial contamination), the number of genome copies of NoV in oysters is usually close to the limit of detection. All these considerations highlight the difficulties in interpreting a positive or negative result when using such an approach [37,38]. Based on the findings of this study, it is recommended to extend the purification duration for an effective reduction of NoV contamination in oysters. The shellfish harvest area will be closed for a minimum of 30 days in Western Canada if the Canadian Food Inspection Agency (CFIA) detects NoV in any single oyster sample. Prolonged purification allows natural shellfish digestion and excretion to reduce viral content more comprehensively. Meanwhile, regular monitoring of water quality levels is essential during the purification process to ensure a controlled and safe environment. The Canadian Shellfish Sanitation Programme (CSSP) manual is a reference document for monitoring, classifying, and controlling areas where bivalve molluscan shellfish are harvested. It is necessary to detect oyster quality during transportation, storage, and processing. Standardization for prolonged purification in the oyster aquaculture industry is of paramount significance. This proactive approach aims to optimize the purification process and enhance its efficiency in mitigating NoV contamination risks in oysters.

Furthermore, the research has certain limitations due to time, energy, and constraints. The NoV extraction method, according to ISO 15216, was designed for digestive gland tissues. The reliability and validity of the gills and palps may not be equally reliable for different oyster tissues. Results should also be interpreted with caution due to the methodological limitations of primary studies, which are reflected in the low certainty of the evidence rating since only oyster tissue changes were considered and not shifts in temperature regimes, oyster species, or NoV genotypes. Some studies show that low temperatures weaken shellfish metabolism [39]. There were significantly higher viral loads among oysters from cold waters (5 °C) than those from warmer waters (>10 °C) [40]. Based on that, it sheds some light on the traditional oyster purification procedures and suggests that temperature might be considered a regulating method. Our study was conducted at room temperature. A further step towards understanding oyster spatial distribution and enrichment dynamics with NoV is to consider temperature as a variable. But again, more artificially contaminated experiments and actual field data are needed to confirm this.

## 5. Conclusions

To conclude, this study focused on the spatial distribution and enrichment dynamics of NoV within different oyster tissues, shedding light on crucial factors influencing the purification process. Various tissues, including the gills, palps, stomach, and digestive glands, were monitored to discern NoV contamination kinetics. The results offer a thorough assessment of NoV contamination in oysters, providing valuable data for the development of efficient purification strategies and detection methods. The findings contribute to ongoing efforts to improve food safety, particularly concerning oyster consumption, and emphasize the significance of refined purification techniques to reduce the dangers associated with NoV.

## Data Availability

Data is contained within the article and supplementary material.

## Supplementary Materials

The following supporting information can be downloaded at: https://www.mdpi.com/article/10.3390/foods13010128/s1, Table S1: The original data of quantitative real-time PCR (RT-qPCR) for Norovirus.
