# Supplementary material for: Spatial Distribution and Enrichment Dynamics of Foodborne Norovirus in Oyster Tissues
Source: Foods. 2023 Dec 29;13(1):128. doi: 10.3390/foods13010128 (PMC10778453; doi:10.3390/foods13010128)
Supplement: Supplementary file 1 [file foods-13-00128-s001.zip › foods-2775663-supplementary.pdf]

Table S1. The original data of quantitative real-time PCR (RT-qPCR) for Norovirus

Ct value	Exposure time	6h	12h	24h	48h	72h	96h
Issue	Parallel samples						
Gill	1	36.63	32.31	32.72	NA	NA	NA
	2	34.09	33.05	32.51	NA	NA	NA
	3	35.75	32.98	35.73	NA	NA	NA
	4	34.98	33.65	32.61	NA	NA	NA
Palp	1	33.72	NA	NA	NA	NA	NA
	2	34.45	NA	NA	NA	NA	NA
	3	35.06	NA	NA	NA	NA	NA
	4	34.02	NA	NA	NA	NA	NA
Stomach	1	35.1	NA	NA	34.74	NA	NA
	2	33.8	NA	NA	37.38	NA	NA
	3	38.8	NA	NA	33.73	NA	NA
	4	35.06	NA	NA	33.65	NA	NA
Digestive gland	1	35.29	32.72	33.84	31.86	33.51	34.83
	2	NA	32.50	33.96	31.95	33.54	34.74
	3	34.98	34.77	33.7	31.58	33.63	34.61
	4	38.28	33.52	33.47	31.49	34.30	34.70
